# Exploratory fNIRS Study of Prefrontal Activation in Drug-Naïve ADHD Across Developmental Stages

**DOI:** 10.1192/j.eurpsy.2026.10569

**Published:** 2026-07-31

**Authors:** S. Lee, W. S. Kang, M. Hong

**Affiliations:** 1 Psychiatry, Kyung Hee University Hospital; 2 Psychiatry, Kyung Hee University College of Medicine; 3 Psychiatry, Kyung Hee University Hospital at Gangdong, Seoul, Korea, Republic Of

## Abstract

**Introduction:**

Attention-deficit/hyperactivity disorder (ADHD) is associated with alterations in executive function and prefrontal cortical activity. Functional near-infrared spectroscopy (fNIRS) provides a non-invasive method to assess task-related hemodynamic changes, yet developmental differences in drug-naïve ADHD populations remain insufficiently understood.

**Objectives:**

This study aimed to investigate prefrontal activation patterns during executive function tasks in drug-naïve individuals with ADHD compared with healthy controls, and to explore age-related differences across childhood, adolescence, and adulthood.

**Methods:**

We enrolled a total of 48 drug-naïve patients with ADHD (children, adolescents, and adults) and 50 healthy controls between March 2019 and February 2022. Participants were recruited from the outpatient clinic of the Child and Adolescent Psychiatry Department. All participants underwent standardized assessments, including IQ testing, ADHD symptom rating scales, and the Computerized Attention Test (CAT). Prefrontal cortical activation was measured using functional near-infrared spectroscopy (fNIRS) during the Trail Making Test A and B. Group comparisons were conducted between ADHD and control participants, as well as across age groups. The study was approved by the Ethical Committee (No. MJH 2010-08-025-001).

**Results:**

ADHD participants showed significantly higher symptom severity compared with controls (all *p* < 0.001), as well as lower performance on the Computerized Attention Test (CAT) (*p* = 0.009) and reduced working memory and processing speed indices of IQ (*p* < 0.01) (Fig 1). fNIRS analyses during the Trail Making Tests revealed distinct activation patterns across groups and age strata.(Fig 2) While overall group-level effects were limited, exploratory ROI-level analyses indicated a significant difference in oxygenated hemoglobin response at ROI4 under specific task conditions, suggesting localized prefrontal hypoactivation in ADHD (Fig 3).

**Image 1:**
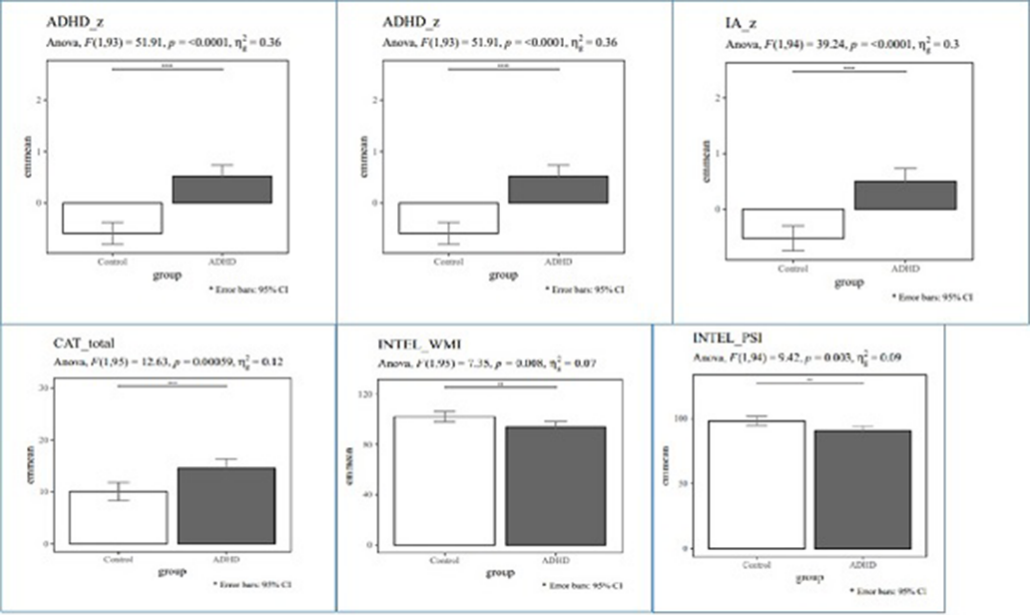
Image 1: Long description.

**Image 2:**
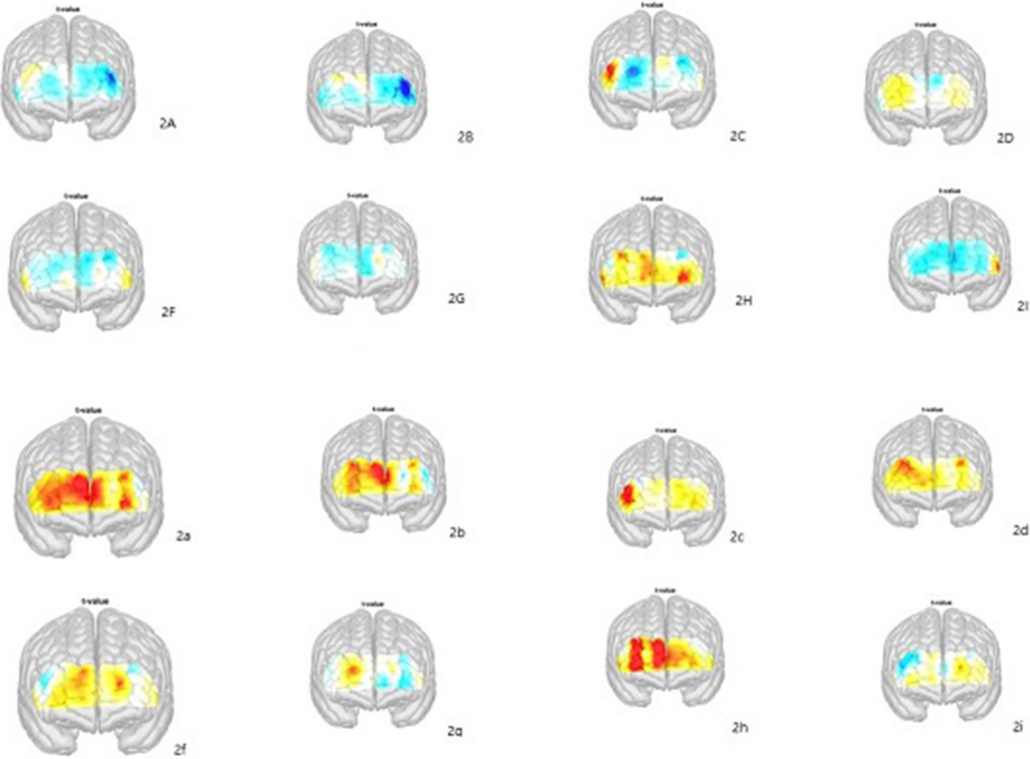
Image 2: Long description.

**Image 3:**
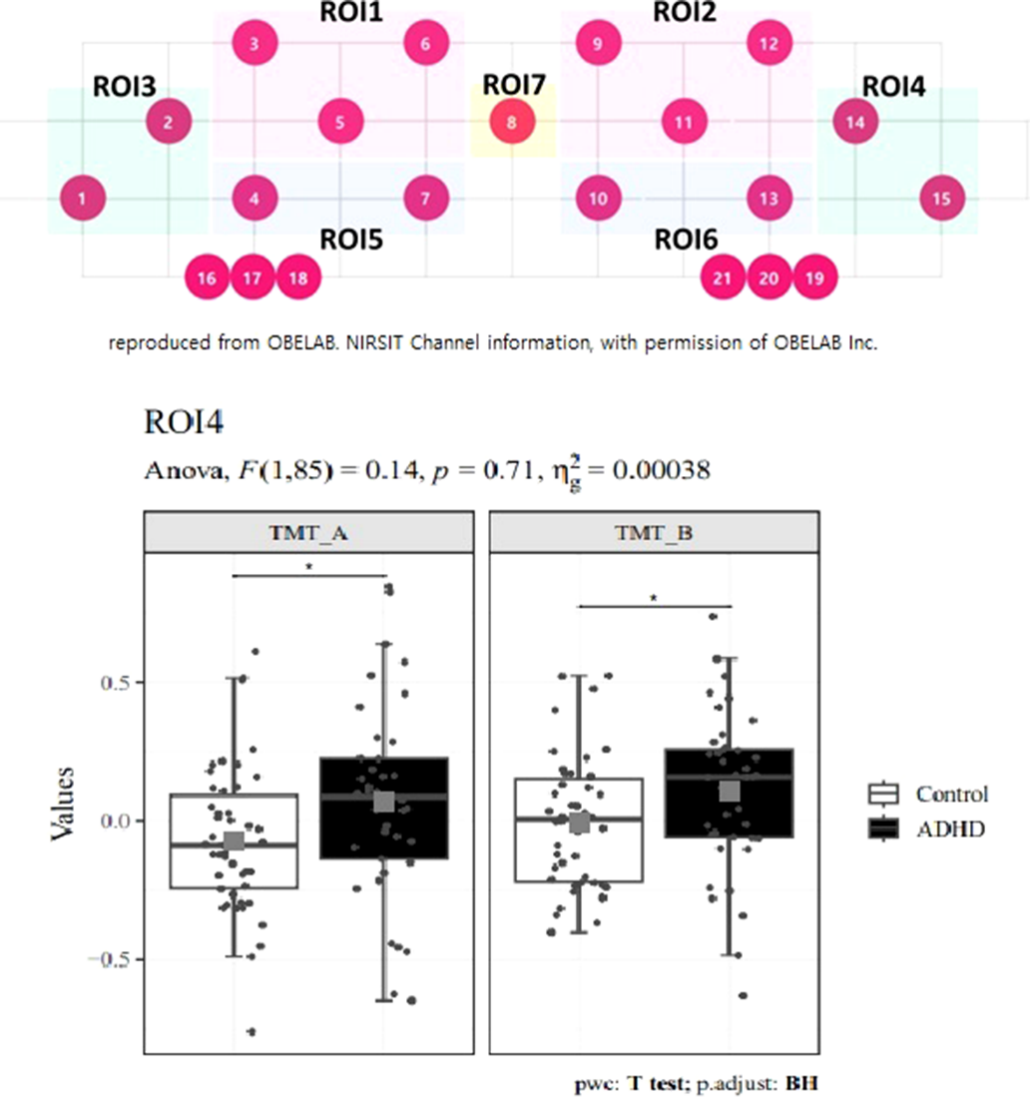
Image 3: Long description.

**Conclusions:**

Drug-naïve individuals with ADHD demonstrated marked clinical and cognitive impairments compared with controls. Although global group differences in prefrontal activation were subtle, the presence of localized alterations such as ROI4 highlights the potential of fNIRS to capture specific neural dysfunctions in ADHD. These findings underscore the importance of developmental context and support the need for larger-scale studies to validate ROI-specific effects.

**Disclosure of Interest:**

S. Lee: None Declared, W. S. Kang: None Declared, M. Hong Grant / Research support from: Supported by National Research Foundation of Korea

## Long descriptions

### Image 1: Long description

The six bar charts are arranged in two rows of three. Each chart plots ‘group’ on the x-axis (Control in white, A D H D in dark gray) against ‘emmean’ on the y-axis, with error bars representing 95% C I.

Top Row:

1. A D H D underscore z: Control is below 0, A D H D is approximately 0.5. Anova, F(1,93) = 51.91, p < 0.0001, eta squared sub g = 0.36.

2. A D H D underscore z (duplicate): Identical to the first chart.

3. I A underscore z: Control is approximately -0.5, A D H D is approximately 0.5. Anova, F(1,94) = 39.24, p < 0.0001, eta squared sub g = 0.3.

Bottom Row:

4. C A T underscore total: Control is at 10, A D H D is approximately 15. Anova, F(1,95) = 12.63, p = 0.00059, eta squared sub g = 0.12.

5. I N T E L underscore W M I: Control is approximately 100, A D H D is slightly lower at approximately 90. Anova, F(1,95) = 7.35, p = 0.008, eta squared sub g = 0.07.

6. I N T E L underscore P S I: Control is approximately 100, A D H D is approximately 90. Anova, F(1,94) = 9.42, p = 0.003, eta squared sub g = 0.09.

All charts show significant differences indicated by horizontal bars with asterisks above the data columns.

Navigate back to Image 1:.

### Image 2: Long description

A multi-panel grid of 16 anatomical brain models viewed from an anterior perspective, focusing on the prefrontal cortex. Each scan is labeled with a t-value indicator at the top and an alphanumeric code at the bottom right. The panels are organized as follows.

Top row panels 2 A, 2 B, 2 C, and 2 D. Panel 2 A and 2 B show concentrated blue cool-toned regions in the right hemisphere. Panel 2 C shows a mix of red warm-toned and blue cool-toned spots. Panel 2 D shows predominantly yellow and orange warm-toned regions across both hemispheres.

Second row panels 2 F, 2 G, 2 H, and 2 I. Panel 2 F and 2 G show light blue and yellow clusters. Panel 2 H displays intense red and orange activation across the frontal lobe. Panel 2 I shows a central blue cluster with a small red spot on the far right.

Third row panels 2 a, 2 b, 2 c, and 2 d. Panel 2 a shows a large, intense red and orange area spanning the left and central frontal regions. Panel 2 b shows similar warm tones concentrated centrally. Panel 2 c and 2 d show smaller, more isolated yellow and red clusters.

Bottom row panels 2 f, 2 g, 2 h, and 2 i. Panel 2 f shows yellow and orange clusters in the lower frontal region. Panel 2 g shows small, faint yellow and blue spots. Panel 2 h shows a dense vertical band of red and orange in the left hemisphere. Panel 2 i shows scattered light blue and yellow spots.

Navigate back to Image 2:.

### Image 3: Long description

The top panel displays a grid layout of 21 numbered pink circular channels organized into seven Regions of Interest, labeled R O I 1 through R O I 7. R O I 3 is on the far left, R O I 4 is on the far right, and R O I 7 is at the center. R O I 1 and R O I 2 are at the top, while R O I 5 and R O I 6 are at the bottom.

The bottom panel focuses on R O I 4 with a statistical header: Anova, F(1,85) = 0.14, p = 0.71, eta super 2 sub g = 0.00038. Below this are two side-by-side box plots labeled T M T underscore A and T M T underscore B.

* In the T M T underscore A plot, the y-axis represents Values from -0.5 to 0.5. It compares a white box (Control) and a black box (A D H D). Both boxes are centered near 0.0, with the A D H D group showing a slightly higher median and wider distribution of individual data points.

* In the T M T underscore B plot, the distribution is similar, with the A D H D group again showing a slightly higher median than the Control group.

* A legend on the right identifies the white box as Control and the black box as A D H D.

* A footer at the bottom right reads: p w c: T test; p dot adjust: B H.

Navigate back to Image 3:.

